# AGEISM FIRST AID: AN ONLINE COURSE DESIGNED TO ADVANCE GERONTOLOGY AND COMBAT AGEISM WITHIN THE HELPING PROFESSIONS

**DOI:** 10.1093/geroni/igz038.185

**Published:** 2019-11-08

**Authors:** Laurinda Reynolds, Tina M Kruger, Rebekah Knight

**Affiliations:** 1 American River College, Sacramento, California, United States; 2 Indiana State University, Terre Haute, Indiana, United States; 3 University of North Texas, Denton, Texas, United States

## Abstract

Ageism First Aid is an online course designed to combat ageism, advance Gerontology, and generate revenue for Gerontology programs and GSA/AGHE activities. The project was funded by a grant from the Retirement Research Foundation and sponsored by the AGHE Academic Program Development Committee. The AFA course content is informed by developmental, social, and cognitive psychology, sociocultural and cognitive linguistics, speech-language science, and geriatrics. The content cultivates ageism awareness by replacing the common misconceptions about aging that underlie ageism with facts. The content also cultivates cultural consciousness and uses concise common language accessible to learners of diverse backgrounds. During this symposium, participants will be introduced to the course content and explore the benefits of this multidisciplinary approach to curriculum development. The results summary of the AFA course pilot will be presented as evidence of the course efficacy. Participants will leave able to utilize AFA within their institutions and local Aging Networks.

